# Solution of underdetermined systems of equations with gridded a priori constraints

**DOI:** 10.1186/2193-1801-3-145

**Published:** 2014-03-17

**Authors:** Stathis C Stiros, Vasso Saltogianni

**Affiliations:** Department of Civil Engineering, University of Patras, Patras, 26500 Greece

**Keywords:** Least squares, Free net adjustment, Singular matrix, Defect, Uncertainty, Deterministic, Stochastic

## Abstract

The TOPINV, Topological Inversion algorithm (or TGS, Topological Grid Search) initially developed for the inversion of highly non-linear redundant systems of equations, can solve a wide range of underdetermined systems of non-linear equations. This approach is a generalization of a previous conclusion that this algorithm can be used for the solution of certain integer ambiguity problems in Geodesy.

The overall approach is based on additional (a priori) information for the unknown variables. In the past, such information was used either to linearize equations around approximate solutions, or to expand systems of observation equations solved on the basis of generalized inverses. In the proposed algorithm, the a priori additional information is used in a third way, as topological constraints to the unknown n variables, leading to an R^n^ grid containing an approximation of the real solution.

The TOPINV algorithm does not focus on point-solutions, but exploits the structural and topological constraints in each system of underdetermined equations in order to identify an optimal closed space in the R^n^ containing the real solution. The centre of gravity of the grid points defining this space corresponds to global, minimum-norm solutions. The rationale and validity of the overall approach are demonstrated on the basis of examples and case studies, including fault modelling, in comparison with SVD solutions and true (reference) values, in an accuracy-oriented approach.

## Introduction

Redundant systems of non-linear equations with n variables and m measurements (m > n) are frequent in various fields of science and engineering, but there does not exist a unique or general method for their solution. In the case of various non-linear problems, such as those arising from observations of distances and angles, as in various fields of Geodesy, algebraic solutions are obtained on the basis of linearization of the observation equations leading to a system of equations (Mikhail 1976). In the case of highly non-linear systems, however, this is not possible, and either certain observation equations are selected to solve a non-redundant system (Ren and Hong 2009), or various numerical/statistical, usually Monte Carlo-based approaches (or genetic algorithms, especially PSO and annealing simulations, Pedersen et al. 2003; Li 2009; Voglis et al. 2012) are used. Some limitations of these techniques are that they usually ignore the error properties (uncertainties) of observations and of the solutions, and they may be trapped in local solutions (see Saltogianni and Stiros 2012a).

A topological inversion technique for the solution of redundant systems of non-linear equations with n unknowns has recently been presented by Saltogianni and Stiros (2012b;^2013^) and was further assessed by Harvey (2013). This technique, thereafter called TOPINV (from Topological Inversion, or TGS, Topological Grid Search), exploits the power of modern computers and is based on the principle of intersection of geometric loci in the R^n^ space. It is a technique inspired from the traditional lighthouse navigations, and one of its major advantages is that it does not require any inversion of matrices. For this reason, it was proposed that this method can also be applied for the solution of certain types of underdetermined systems of equations (Harvey 2013), the solution (inversion) of which traditionally leads to inversion of singular matrices (Matsu’ura and Hirata 1982).

In this article we present a generalization of the idea of Harvey (2013) that TOPINV (or TGS) can cover a wide range of underdetermined systems of equations observed in engineering and geophysics. We also explain that the only requirement for this method of inversion is the a priori knowledge of the possible range of values for each of the unknown variables. This requirement is far from being unusual, and in fact it represents another way to accommodate additional information or external constraints conventionally used for the solution of underdetermined systems of equations (Matsu’ura and Hirata 1982; see “A priori information used for the inversion”).

Several examples and case studies are presented and permit to validate the results of this method on the basis of surrogate (synthetic) data, in comparison to SVD-based solutions. This is an accuracy-oriented validation, based on the comparison of a priori known (“true”) solutions of a system of equations with that deriving from the TOPINV (or TGS) algorithm.

### The TOPINV method

The TOPINV (or TGS) algorithm is fully explained in Saltogianni and Stiros (2012b;^2013^) and is summarized in this section.

**Figure 1:**
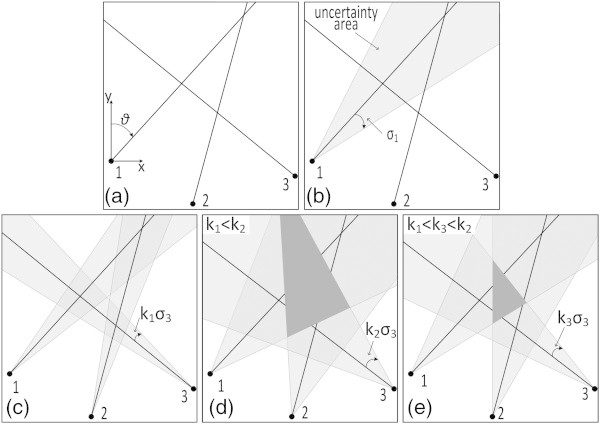
**Schematic representation of the TOPINV inversion in the case of intersection of three azimuth lines. (a)** Because of measurement errors (shaded areas), the three azimuths (directions relative to the north) do not converge into a single point. **(b)** Introducing an uncertainty angle based on the standard errors *σ*, each of these areas corresponds to an uncertainty area *S*_*j*_. **(c-e)**. Using a scale factor *k*, the optimal common section *S* of areas *S*_*j*_can be obtained.

Let us assume a system of m (non-linear) equations *f*_*j*_, *j* = 1, 2, …, m with n unknowns *x*_*i*_, *i* = 1, 2, …n
1

where ℓ_*j*_ indicates a measurement with standard deviation *σ*_*j*_ and *υ*_*j*_ an unknown random error. These equations are not mutually consistent because of errors in measurements and imperfections of the model adopted; this is schematically shown for three observations of azimuths in Figure 1a.

In conventional algebraic (least squares) the point solution is obtained on the basis of minimization of weighted squares of *υ*_*j*_, but this requires an inversion of linearized equations (Mikhail 1976) which is possible in the case of redundant systems only.

The TOPINV method is based on two considerations.

*First*, on a priori constraints for the solution of a system, i.e. that the possible values (solution) of each unknown variable *x*_*i*_ are subject to the conditions
2

with *x*_*i,min*_, *x*_*i,max*_ known values, and that the above range of possible values can be approximated by a series (sets) of discrete, equally spaced points.

For the n variables, these sets of points define an n-dimensional grid G which defines a closed space containing all possible solution of the system of equations; some of the grid points approximating the solution of the system of Eq. (1).

*Second*, in order to overcome the problem of non-mutually consistent observation equations (Figure 1), each equation (1) is transformed into an inequality
3

Forward computations and Boolean logic permit to identify which points of G (in fact a set *S*_*j*_ of gridpoints) satisfy Eq. (1), i.e. to identify the geometric locus *S*_*j*_ of the solution of this equation. If a certain point M represents the solution of the system of equations Eqs (1), it must satisfy all inequalities (3) for j = 1,2, … m, and it will be located in the intersection *S* of all areas *S*_*j*_.
4

It is possible this intersection to be very large (Figure 1d), or not even exist (Figure 1c). This problem can be overpassed introducing a scale (optimization) factor *k* according to the equation
5

This scale factor *k* is determined empirically (with trials) and permits to shrink or expand the uncertainty margin of each observation (shown as an angle, highly exaggerated in Figure 1) and of *S*_*j*_ and *S*, until a minimum (optimal) common intersection, i.e. until a minimum space *S* containing the solution of the system of equations is obtained (Figure 1e). The overall approach is described in Figure 1 for two variables, i.e. in an R^2^ space, but it can be generalized for n variables, i.e. for a grid G in the R^n^ (n-D space).

By definition, the set of grid points *S* represents a space containing the real solution. The centre of gravity of the grid points of set *S* (first moment of the population of these grid points included in set *S*) defines statistically a very good (minimum bias) estimator  of the true solution  of the system of observation equations, i.e.


and from the population of its grid-points, it is easy to compute the variance-covariance matrix of the estimated solution.

This is valid only if the n-D space *S* is compact, convex. If not, this is indicative of different solutions, and in this case, *S* should be split into sub-spaces, each providing an independent solution.

Other practical problems may arise, for instance a grid too large, requiring too much computer time. In this case, a large and coarser grid G is selected first, a space *S* is identified, and then a smaller and finer grid around *S* is used to refine the solution each providing a different solution.

This technique has several main advantages, especially that it does not require inversion of matrices, it is not focusing on point solutions, and hence the solution is not trapped into local maxima/minima (see Saltogianni and Stiros 2012a) and it is free of the limitations of the various sampling techniques (see Li 2009), because it is based on a deterministic analysis of the whole grid G.

### Underdetermined systems of observation equations

Underdetermined systems of observation equations are of different types, and their classification can be easily made on the basis of visualized, simple geometric (geodetic) observation systems, i.e. of systems of observations of angles and of distances in a 2-D space, as is explained in Example 1. The solution of such underdetermined systems is usually based on SVD techniques, but the quality of the corresponding solutions depends on the initial conditions (Example 2). Alternative techniques, such as Bayesian statistics have also been used (Zhu et al. 2001).

#### Example 1

**Figure 2:**
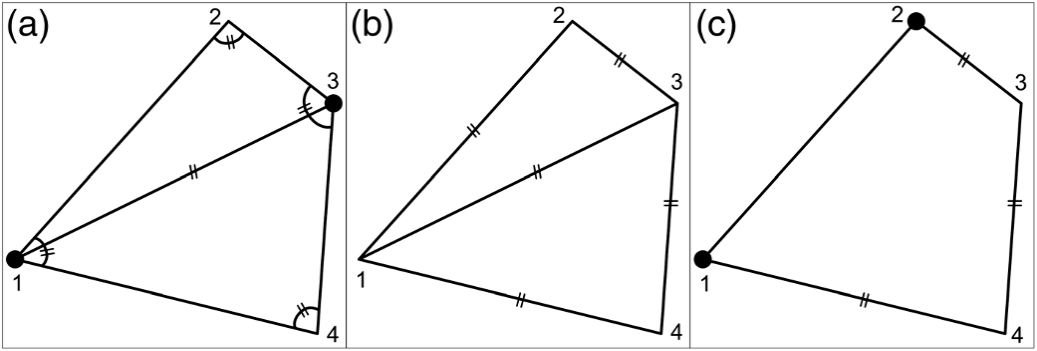
**Geometric visualization of over-determined systems and of systems with datum defect or configuration defect. (a)** over-determined systems **(b)** systems with datum defect or **(c)** with configuration defect. Two small lines indicate measured angle or distance, solid circles points of known coordinates.

Figure 2a shows a quadrangle of which there have been measured the angles and one diagonal, while the coordinates of two of the corner points are known. The available data permit to define a redundant system of non-linear equations combining observations (angles and lengths) with the coordinates of four known and unknown points. This system can be solved using typical least squares techniques, after the equations are linearized (Mikhail 1976). As will be emphasized later, this linearization requires a certain a priori knowledge/constraint/condition that the solutions are in the vicinity of a priori known approximate solutions.

In Figure 2b only the length of the sides and one diagonal of a quadrilateral have been measured, and no coordinates are known. The available data permit to define the geometry (shape and dimensions) of the quadrilateral, but the coordinates of its corner points cannot be computed. This example is representative of a large category of underdetermined systems of equations, reflecting a *datum defect*. In the past, this defect was usual before the advent of GPS in tectonics studies, because the available geodetic observations of distances and of angles did not permit estimations of absolute displacements. Still, the addition of some constraints (additional information for coordinates or for fault-slip) permitted to overpass the datum defect (Brunner 1979; Prescott 1981).

In Figure 2c, the coordinates of two adjacent points of a quadrilateral are known, and only the lengths of the three sides have been measured. Hence the coordinates of the two remaining corners cannot be defined, because the available data do not permit to constrain the shape of the quadrilateral; it corresponds to a mechanism. The system of observations cannot hence been solved, because an observation necessary to constrain the shape of the quadrilateral is missing. This leads to a geometry/shape not defined, and to a *configuration defect* (or to singular configuration).

No solution for such cases is readily available. Such a defect may be found in any field of science and engineering. An example: an earthquake recorded in only one seismogram, permits to compute only the distance between the epicentre and the seismogram, but not the epicentre; the latter is typically defined as the intersection of two geometrical loci, of two circles defined by the computed distances of the epicentre from two seismological stations. Singular configurations in robot manipulators (Sokolov and Xirouchakis 2006) are another example.

In certain common special cases the configuration defect derives from a *scale ambiguity*, for instance from a triangle in which only angles have been measured. An additional information (constrain, hypothesis) for a length is necessary to remove this defect (ambiguity); this is the case of the analysis of traditional triangulation data (Stiros 1993). In other cases a certain type of configuration defect derives from an *integer ambiguity*. Such an ambiguity derives from phase measurements of a wave of certain wavelength λ emitted from an instrument, reflected on a certain surface and then received back by the instrument. In this case the distance s can be computed from the equation
6

where n is an unknown integer and φ the measured phase between emitted-received wave. Such integer ambiguities represent a major source of error in GPS positioning (Han and Rizos 1996), but also in satellite radar measurements (Usai 2003; Kampes and Hanssen 2004) etc. As has been shown by Harvey (2013), certain of these problems can be solved on the basis of the TOPINV.

From the analytical point of view, any configuration or datum defect leads to a certain singular matrix which cannot be inverted, and this problem is usually solved on the basis of generalized matrix inverses and especially the Single Value Decomposition (SVD) technique (Matsu’ura and Hirata 1982; Strang 2003). The overall significance of SVD is that it identifies the best solution which satisfies observations. If certain conditions are satisfied, SVD permits optimal solutions and this explains its application in a large number of studies in different fields of sciences and engineering. The limitations and requirements for a successful SVD solution are explained in the following Example 2.

#### Example 2

Let us assume that there has been measured the height difference *h* = 2.1 (arbitrary units) between two points A, B with elevations *z*_*A*_ = 4 and *z*_*B*_ = 2 but unknown to the observer. This leads to the equation
7a

with *υ* indicating an unknown observation error as in Eq. (1). This equation can be written in matrix form
7b

This system of one equation is rank defect because of a datum defect, it leads to an infinity of solutions, and typically cannot be solved. The SVD solution, however, leads to a minimum norm solution, *z*_*A*_ = *h*/2 = 1.05, *z*_*B*_ = - *h*/2 = - 1.05.

Clearly, this solution is different from the real values of *z*_*A*_ and *z*_*B*_. If an a priori additional information (constraint) is available, for example that the approximate elevations of the two points A and B are H_A_ = 4.1 and H_B_ = 1.9, Eq. (7a) becomes
8a

where *δ*_*A*_, *δ*_*B*_ are the unknown differences between real and approximate elevations of A and B. Hence, Eq. (8a) takes the form
8b

and the corresponding SVD solution would be
9

These new estimates tend to the real values of elevations of A and B if the approximate estimations H_A_ and H_B_ were very close to the real values. Eqs. (9) indicate that the accuracy of the SVD-derived solution depends on the accuracy of approximate values and the noise of measurements (cf. Xu 1998). The overall approach of course can be easily generalized to more variables and observation equations.

### A priori information used for the inversion

The solution of the systems of observation equations depends on their type, linear or not, and redundancy, and is based either on algebraic or numerical techniques (Mikhail 1976; Kaipio and Somersalo 2005; Tarantola 2005; Vogel 2002).

The algebraic solution of certain systems of redundant, non-linear equations (adjustment in Geodesy) is based on the linearization of equations using Taylor’s formula and the application of the least squares criterion which permits to solve systems of linear equations of the form
10

(Mikhail 1976), the linearization of these equations is, however, not unconditional, and has a meaning *only* in the vicinity of the true position  (“ideal” solution) of an unknown variable *x*, i.e.
11

where , , **ϵ** and **0** are n-dimensional vectors, **0** a zero vector, and each component of **ϵ** can be regarded as a random variable with zero mean and variance σ, i.e. with statistical distribution (0, *σ*^2^); otherwise the linearization is not valid. Condition (11) hence represents an a priori additional information or an external constraint, necessary for the solution of non-linear systems of equations. This a priori or additional information is classified as the *first type* of a priori information.

Certainly, approximate solutions can in many cases be obtained from preliminary solutions of the system of equations (for instance selecting a number of equations). Still, this is possible only in the case of relatively simple equations, such as observations of distances or angles, usually in geodetic applications, or in the cases of iterative, converging solutions (Schaffrin and Wieser 2011). On the contrary, in the cases of highly non-linear, redundant systems of equations met in various geophysical problems, preliminary or iterative solutions may lead to local solutions (local minima) very different from the real (global) solution (see figure thirteen in Saltogianni and Stiros 2012a) and hence the conditions of linearization are not met.

Any algebraic solution of Eqs (1) requires the inversion of a certain matrix (Mikhail 1976; Kotsakis 2012). If the system of observation equations is not well-determined, this matrix is rank-defect and a formal least-square solution (i.e. a unique solution) is not possible. However, in some cases of singular matrices, a single (optimal) solution is possible using additional information which removes the rank defect and permits a unique solution, though at the risk of biased results (cf. Usai 2003).

In most cases this additional information is incorporated in the system of equations, for instance pseudo-equations (Kampes and Hanssen 2004), hypotheses for the coordinates or for displacement vectors (Brunner 1979; Prescott 1981), or for the statistical characteristics of some variables, in the case of a hypothesis for Bayesian statistics (Jackson and Matsu’ura 1985; Zhu et al. 2001). These approaches are usually based on generalized matrix inverses (Bjerhammar 1973; Matsu’ura and Hirata 1982). This approach is known in Geodesy as free net adjustment and is discussed by Brunner (1979), Prescott (1981) and recently by Kotsakis (2012) who includes an extensive literature on this topic. This is indeed a *second type* of additional information (or of additional conditions) imposed on a system of equations in order to obtain its algebraic solution.

TOPINV (TGS) introduces a *third type* of additional/a priori information that can be used for the solution of systems of equations. This information corresponds to constraining the expected solution of each of the unknown variables to a certain range of possible values and defining a grid G in the R^n^ space. This grid G is then used for the application of the TOPINV algorithm (see section “The TOPINV method”).

The physical significance of this type of a priori constraints is in some cases evident: the epicentre of an earthquake should be inside the earth, in a certain range of depths, in most cases a GPS receiver can only be on or near the ground surface, etc.

### Methodological approach

#### Internal (structural and geometric) constraints in defective systems

The basic characteristic of underdetermined systems is that they can accept an infinite number of solutions. However, such possible solutions are not randomly distributed and are dominated by certain internal constraints, structural and geometric. For instance, the locus of the foot of the moving leg of a robot is usually a sphere, with centre at the joint of the leg and radius equal to its length (a geometric locus). Additional geometric constrains (another geometric locus, a plane, a torus, etc.) permit to define analytically the position of this foot and control the robot motion using systems of equations (Sokolov and Xirouchakis 2006; Ren and Hong 2009). Intersections of geometric loci therefore define structural constraints and this is easily highlighted in the following example.

#### Example 3

**Figure 3:**
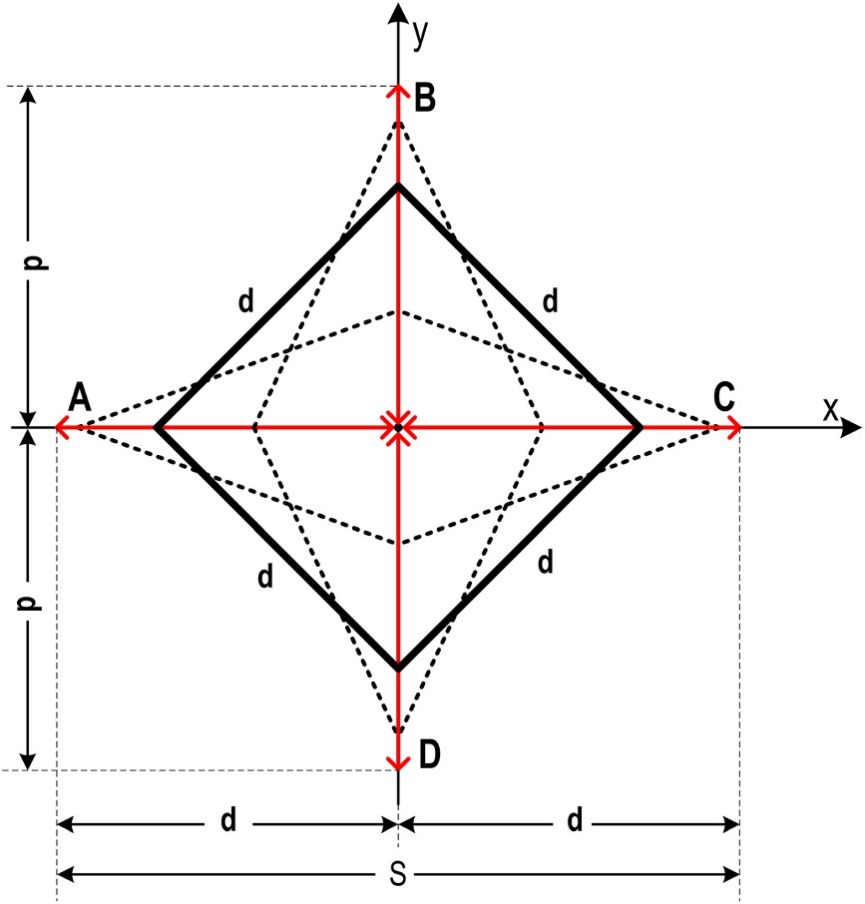
**A mechanism consisting of four equal legs with hinges at their edges.** This mechanism corresponds to a rhomb, shown for two cases (solid and dashed lines). Assuming a fixed centre of gravity at the origin and one of the coordinates on the x-axis, the loci of the edge points are defined. Red lines (segments of length d) indicate the loci of points A, B, C, D. In addition, structural constraints are expressed by Eq. 12.

Let us consider a mechanism consisting of four equal legs of length d with hinges at their edges (Figure 3). This mechanism corresponds to a rhomb ABCD which has one degree of freedom in its configuration, i.e. one observation is missing to unambiguously define its shape and this leads to a configuration defect. In addition, the lack of information in coordinates leads to a datum defect. If the centre of gravity of the rhomb is assumed fixed on the origin of the coordinate system, and it is assumed without loss of generality that one point is located on axis x, the datum defect is removed (see Brunner 1979; Prescott 1981). If in addition a value S of the diagonal is assumed, the configuration defect will be also removed and the coordinates of the corners of the rhomb can be computed.

More explicitly, corners A, B, C, D will be located on axes x and y in positions (coordinates) constrained by the length d of sides and the selected length s of the diagonal, defined by the relationship
12

It can easily be deduced that 0 ≤ S ≤ 2d and as a consequence, for all possible values of S the loci of all corner points are segments of length d along the axes x, y (Figure 3).

This example highlights the fact that in various underdetermined systems their (infinite) solutions are subject to two types of constraints:

First, *geometric (structural) constraints*; in the case of the mechanism of Figure 3 this constrain is expressed by Eq. (12).

Second, *topological (location) constraints*, expressed by the geometric loci of the variables of the system.

These constraints are not always clear, especially in complicated systems or systems with several degrees of freedom and several variables. Still, the situation is clarified in the following Example 4.

#### Example 4

**Figure 4:**
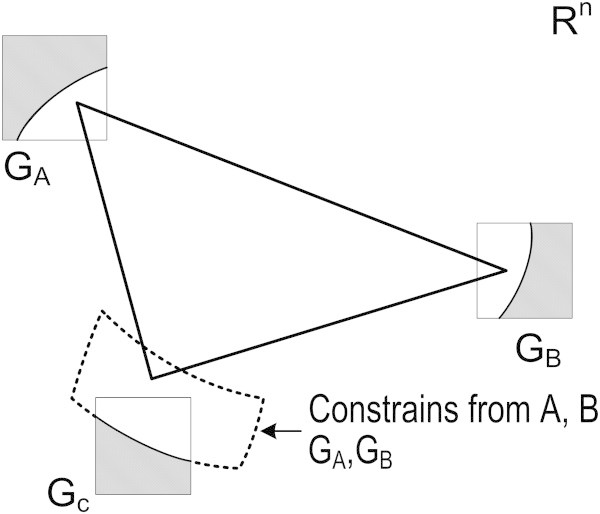
**A triangle of known shape with information about the possible location of its vertices (square areas).** The existing geometric (structural) constraints for the triangle permit to discard certain parts of the squares as possible locations of A, B, C (shaded areas) and define topological constraints for these points.

Three sides of a triangle ABC have been measured, fully constraining its configuration (structural constraints). No information on the coordinates of the triangle exist (datum defect) and hence the system of observation equations formed by the three length measurements has an infinite number of solutions (Figure 4). It is assumed that there exists a priori additional information about the location (coordinates) of points A, B and C, for simplicity shown as squares. The traditional approach is to use these equations as additional equations in the system of observations, and solve this system using a conventional generalized matrix approach (Kotsakis 2012).

An alternative approach is to use the additional information for the coordinates of the three points as topological constraints of the unknown variables, as is highlighted in Figure 4. In this Figure, the possible locations of each point are shown by squares G_A_, G_B_, G_C_. The three points A, B, C should form a triangle of specific shape (structural constrain). This means that if point A is constrained to grid G_A_, point B can be only in certain parts of grid G_B_, and vice-versa. Hence some parts of these two grids can be discarded as possible locations of these points (shaded areas). The possible combinations of coordinates of A and B for the same reason permit to discard some parts of grid G_C_. The role of TOPINV is indeed to identify and exploit the critical combination of the geometry of the triangle (structural constraint) and of the loci of points A,B,C (topological constraints).

#### Alternative solution of underdetermined systems of equations

Among the (infinite) possible solutions for the system of Figure 3, an unconstrained SVD would lead to a solution with equal diagonals, a solution characterized by minimum norm in the differences of the coordinates of the corner points. This solution, however, requires linearization of the non-linear equations, and this requires additional (a priori) information (or constraints) for the unknown variables (coordinates).

We shall show that a solution to such non-linear problems is possible without any linearization, simply adopting the TOPINV algorithm.

A usual problem is to estimate the unknown coordinates of a point M using measurements of distance from two, three or more points *P*_*j*_ of known coordinates; this is a common problem in conventional Surveying (determining an unknown position using mapping intersection techniques), in Seismology (computation of the epicentre of an earthquake from recordings of seismographic stations) and in Satellite Geodesy (computation of the unknown coordinates of a GPS receiver from the measured distances of the receiver from the known coordinates of satellites).

**Figure 5:**
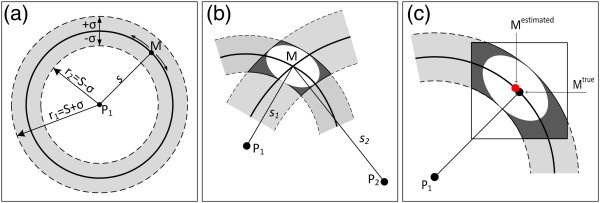
**Uncertainty areas and their intersections. (a)** A ring defines the uncertainty area (locus) of point M, the distance S of which has been measured from point *P*_*1*_with standard error *σ*. **(b)** Schematic representation of the problem of intersection. The coordinates of a point M are defined from the common space (intersection) of the areas of uncertainty of each observation. The best estimate of the coordinates of point M is defined as the centre of the ellipse inscribed in this common area (space). **(c)** Topological estimation of the coordinates of point M as intersection of the area of uncertainty of M (ring) and a square, reflecting an a priori condition for its location. This last area corresponds to the grid G of TOPINV.

The conventional analytical approach can be visualized as determination of the area of uncertainty of each measurement, and then of their intersection. The most probable value of the location (coordinates) or best estimate of M will be at the centre of the ellipse inscribed in this intersection area (Mikhail 1976). In a 2-D space, this of course requires at least two observations of distance to avoid singular matrices (Figure 5b).

In the case of a single observation of distance, a singular matrix is obtained, but an a priori knowledge of coordinates of M can lead to additional observation equations and a redundant system.

A modification of this approach is indeed adopted by TOPINV, as explained below.

The geometric locus of M typically is a circle with centre *P*_1_ (known point) and radius S (distance measurement; Figure 5a). However, measurement S contains errors, assumed for simplicity random with a statistical distribution (0, *σ*^2^). For this reason point M is assumed to be located not in a circle with centre *P*_1_ (locus of M in the Euclidean Geometry), but in a ring (2-D space) bounded by two circles with radii *r*_*1*_ = S + *kσ*, *r*_*2*_ = S - *kσ*, with a probability (statistical significance level) depending on the value of *k* (Mikhail 1976; Figure 5a).

If additional information for the location of M is available, i.e. that it is located in a rectangular of uncertainty, the likely area of location of M will be the intersection of the two loci, of the ring and of the rectangular (cf. Eq. 4; Figure 5c). The centre of gravity of their intersection practically coincides with the Best Linear Unbiased Estimator (BLUE-type estimate) of M. This approach explains the function of the TOPINV algorithm (Saltogianni and Stiros 2012a, b; Harvey 2013). The variance (quality, uncertainty) of the estimator, however depends on the prior information for point M, i.e. the quality (accuracy) of the selected grid G.

These explain that the TOPINV algorithm can be used for the inversion of underdetermined systems because it is based on forward computations only (no need for inversion of singular matrices).

### Case studies

The efficiency of this method is demonstrated in certain problems leading to under-determined systems of equations, both simple geometric, which permit an easy visualization, and geophysical. The solution is checked in comparison to reference (real) values and SVD solutions.

### Case study 1: a 2-D linear geometric/survey problem

**Figure 6:**
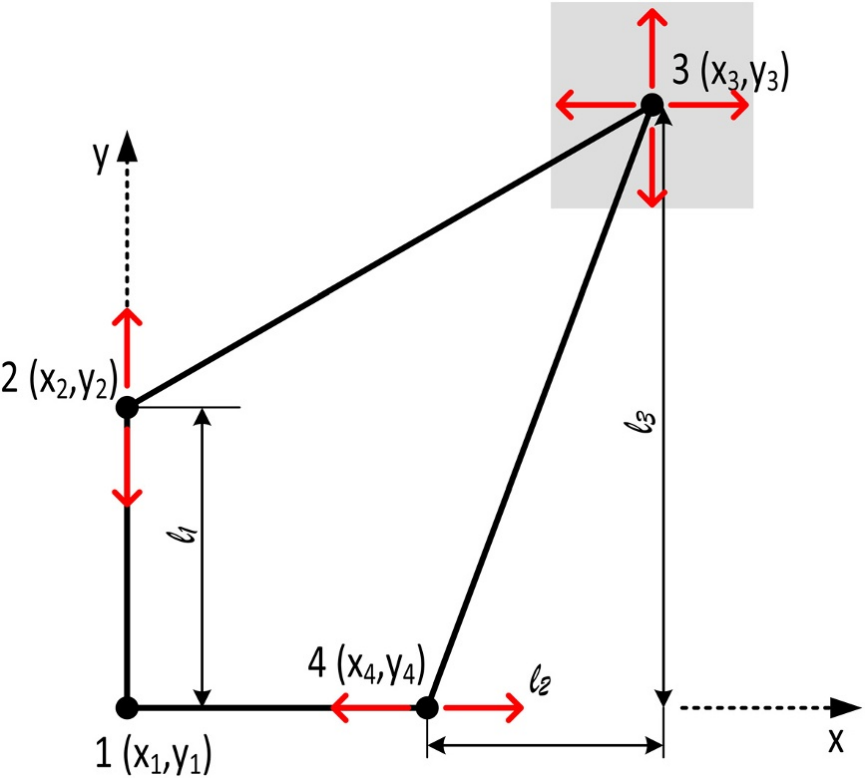
**The 4-point 2-D network examined in Case study 1.** Arrows indicate the degrees of freedom (possible shifts in corresponding axes). Observations ℓ_*j*_are shown. Point 1 corresponds to the origin and points 2 and 4 lie on the y and x axis, respectively.

We examine an underdetermined 2D geometric network consisting of 4 points. For simplicity and without any loss of generality, point 1 was selected as the origin of the coordinate system x-y and points 2 and 4 to lie on axis y and x, respectively (Figure 6).

The technique adopted is the following. We assumed we know the real (reference) coordinates of the four points, and from these coordinates we computed the distances of points lying on the same axis (linear measurements). Adding random noise (*σ*_*j*_ = ±4 mm) there were formed three hypothetical (synthetic, surrogate) measurements of differences of coordinates (*x*_*2*_ - *x*_*1*_ = *l*_*1*_ + *υ*, etc.; Table 1). We then assumed that four coordinates, *y*_*2*_, *x*_*4*_, *x*_*3*_, *y*_*3*_ are unknown, but we a priori know their approximate coordinates, i.e. that they range to ±5 cm from the real (reference) values. These observations lead to a system of observation equations with configuration defect (underdetermined shape, system).

**Table 1:** **Coordinates and synthetic measurements of differences of coordinates in the 2-D network of Case study 1**

Fixed (known) coordinates (m)		Reference (unknown) coordinates (m)		Synthetic measurements (m)	
*x*_1_	0.000	*y*_2_	100.000	ℓ_1_	100.003
*y*_1_	0.000	*x*_3_	200.000	ℓ_2_	99.996
*x*_2_	0.000	*y*_3_	200.000	ℓ_3_	200.001
*y*_4_	0.000	*x*_4_	100.000		

This system was solved first with SVD and then with the TOPINV algorithm, and the results were compared with the reference (true) values.

The four unknown coordinates *y*_*2*_, *x*_*4*_, *x*_*3*_, *y*_*3*_ define a 4-D problem. The additional information of the location (possible values) of these unknown coordinates permits to define a search grid G with characteristics summarized in Table 2.

**Table 2:** **Details of grid G used for the TOPINV inversion of Case study 1**

Coordinates	Reference coordinates	Grid boundaries (m)	Spacing (mm)	Grid points	Total grid points in G
*y*_2_	100.000	99.950–100.050	1.0	101	101^4^ = 104, 060, 401
*x*_3_	200.000	199.950–200.050		101	
*y*_3_	200.000	199.950–200.050		101	
*x*_4_	100.000	99.950–100.050		101	

#### System of equations

The system of equations describing the problem is
13

This is an underdetermined system of equations of the type (10) with *i* = 1,2,…,n = 4 unknown variables and *j* = 1,2,…,m = 3 measurements/equations.

#### SVD solution

At first, an unconstrained SVD solution (i.e. with no a priori constrains for the unknown variables), was readily computed from Eqs. (13) and is marked as SVD1 in Table 3. Some of the computed coordinates significantly deviate from the reference values. For this reason we focused on a SVD solution using additional information. We computed approximate values  of the unknown coordinates adding white noise to their reference (true) values,

**Table 3:** **Comparison of the TOPINV and SVD solutions with the reference values for case study 1**

Reference coordinates (m)		TOPINV ( ***k*** =0.25)		SVD1	SVD2
			± ***σ***		
*y*_2_	100.000	100.003	0.001	100.003	100.003
*x*_3_	200.000	199.998	0.028	**49.998**	199.970
*y*_3_	200.000	200.002	0.001	200.010	200.001
*x*_4_	100.000	100.002	0.028	**-49.998**	99.974

Biased estimates are shown bold. Values in meters.

System (13) was then remodelled on the basis of the equation

14in order to take advantage of the additional constraints and is solved for *δx*_*i*_ using SVD. Then the estimate  of *x* was computed using Eq. (14). Results are summarized as SVD2 in Table 3 and are very close to the reference (true) values of the unknown variables. This is because the approximate values of the unknown variables were selected close to the real values. This is practically the second type of constraints (additional information incorporated in the system of observation equations).

#### TOPINV solution

At first, the system of Eq. (13) was transformed into a system of inequalities (3) in order to account for the stochastic properties of the measurements, and a 4-D grid G with all possible values of vector *x* was formed (Table 2); this grid consists of 101^4^ grid points in total and summarizes the additional information available. Then it was searched which points (4-D vectors) of G satisfy inequality (3) for various values of *k*. The optimal set *S* including the solution of the system of equations for *k* = 0.25 was identified and the centre of gravity of the grid points of *S* and their variances were computed and are shown in Table 3. A close match between estimated and reference (real) values is observed.

### Case study 2: a 2-D nonlinear geodetic/geometric problem

**Figure 7:**
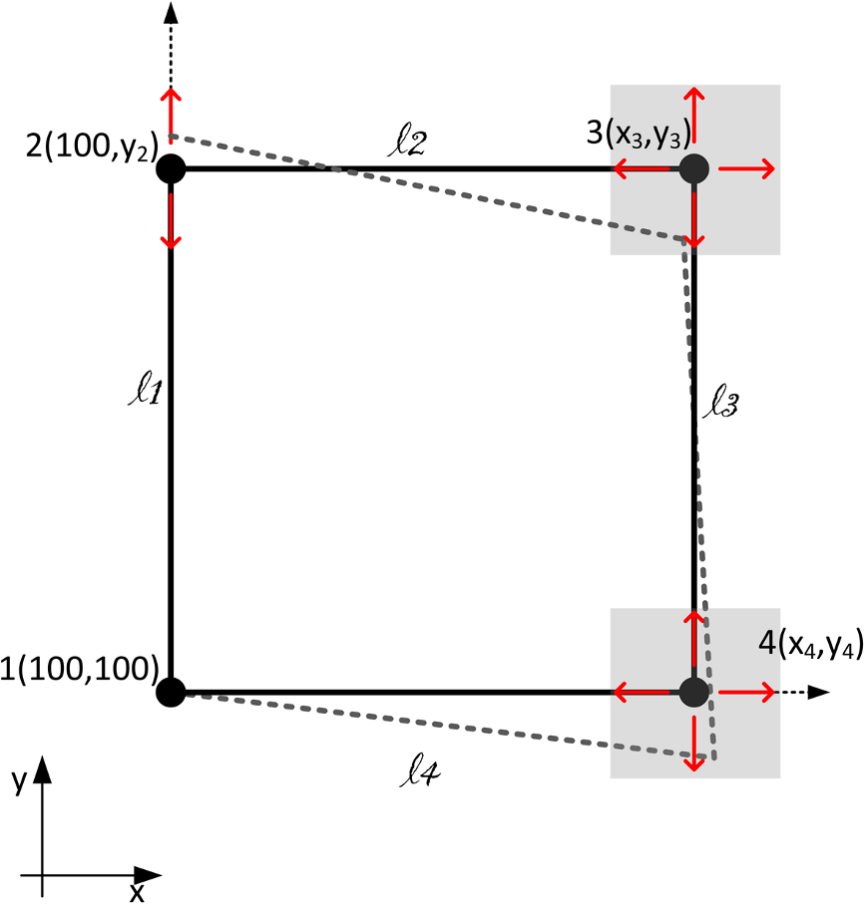
**A 2D geodetic network and the corresponding observations of distances of Case study 2.** Red arrows indicate possible movements of points 2, 3, 4 (degrees of freedom of the system), shaded areas 2-D areas of uncertainty of points. The coordinates of point 1 and the abscissa of point 2 were assumed fixed.

We examine another underdetermined 2D geometric (geodetic) network consisting of 4 points. For simplicity and without any loss of generality, the coordinates of point 1 were assumed known and points 1 and 2 were assumed to share the same abscissa (Figure 7). We assumed known the true (reference) coordinates of the four points defining the quadrangle, and from these coordinates we computed the lengths of its four sides. Adding random noise (*σ*_*j*_ = ±4 mm) four synthetic (hypothetical) measurements were formed (Table 4). These observations lead to an underdetermined system of four non-linear observation equations (*i* = 1,2,…,m = 4) with five unknowns (*j* = 1,2,…,n = 5), i.e. a system with configuration defect. We then assumed that five coordinates, *y*_*2*_, *x*_*3*_, *y*_*3*_, *x*_*4*_, *y*_*4*_ are unknown, but we are priori know that they range to ±2.5 cm from their true (reference) values. The details of the network are described in Table 5. This system was again solved first with SVD and then with TOPINV, and the results were compared with the reference (true) values.

**Table 4:** **Point coordinates and synthetic measurements of distances in the examined 2-D network of case study 2**

Fixed (known) coordinates (m)		Reference (unknown) coordinates (m)		Synthetic measurements (m)	
*x*_1_	100.000	*y*_2_	200.000	ℓ_1_	99.996
*y*_1_	100.000	*x*_3_	200.000	ℓ_2_	100.002
*x*_2_	100.000	*y*_3_	200.000	ℓ_3_	100.003
		*x*_4_	200.000	ℓ_4_	99.999
		*y*_4_	100.000		

**Table 5:** **Details of grid G used for the TOPINV inversion for Case study 2**

Coordinates	Reference coordinates	Grid boundaries (m)	Spacing (mm)	Grid points	Total grid points in G
*y*_2_	200.000	199.975–200.025	1.0	51	51^5^ = 345,025,251
*x*_3_	200.000	199.975–200.025		51	
*y*_3_	200.000	199.975–200.025		51	
*x*_4_	200.000	199.975–200.025		51	
*y*_4_	100.000	99.975–100.025		51	

#### System of equations

The system of four equations with five unknowns describing the problem is symbolically described by Eqs (15)
15

#### SVD Solution

Because the functions *f* in Eqs. (15) connecting unknown variables *x* and measurements ***ℓ*** are nonlinear, the system was first linearized assuming approximate values of *x* deriving from the reference (true) values. Then, based on the linear transformation , system (15) yields the linear system
16

where *J* is the Jacobian of *f*,  a vector of known terms, and **υ** a vector of unknown errors. Eq. (16) was readily solved for *δ*ℓ using SVD and then the estimate  of *x* was computed and is shown in Table 6.  is an unbiased and precise estimator of *x* because  was also an unbiased and precise estimator of *x*. This is a solution corresponding to the first type of accommodation of additional (a priori) information (see section “A priori information used for the inversion”).

**Table 6:** **Comparison of the TOPINV and SVDsolutions with the reference values for Case study 2**

Reference coordinates		TOPINV ( ***k*** =0.25)		SVD
			± ***σ***	
*y*_2_	200.000	199.997	0.001	199.996
*x*_3_	200.000	200.002	0.001	200.002
*y*_3_	200.000	200.001	0.014	199.983
*x*_4_	200.000	199.999	0.001	199.999
*y*_4_	100.000	99.998	0.014	99.980

Values in meters.

#### TOPINV solution

As in the previous Case Study, the system of Eq. (15) was transformed into a system of inequalities (5), and the 5-D grid G with all possible values of vector *x* (51^5^ grid points in total) was formed under the assumption that the possible values of variables are in a range ±2.5 cm around their reference values (Table 5). Then on the basis of the TOPINV algorithm it was searched which set of 5-D points of G satisfy inequalities (5) for various values of *k*. The optimum solution was obtained for *k* = 0.25, and from the set of grid points, their centre of gravity and variances were computed and are shown in Table 6. The computed coordinates are very close to the reference values, and statistically similar.

### Case study 3: a 2-D nonlinear geodetic/geometric problem

**Figure 8:**
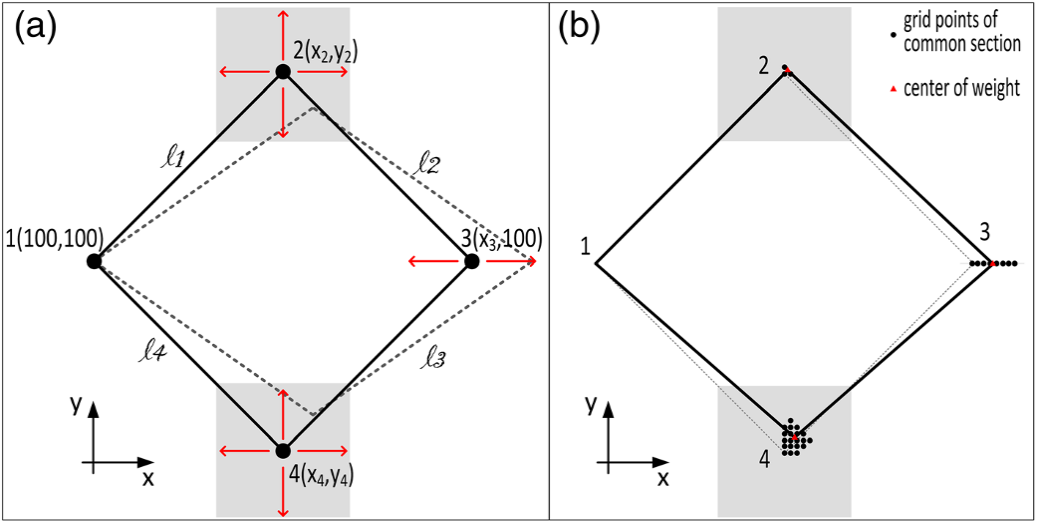
**A 2D geodetic network and the corresponding observations of distances of Case study 3. (a)** Coordinates *x*_*1*_, *y*_*1*_, *y*_*3*_are assumed fixed. Red arrows indicate possible movements of points 2, 3, 4 (degrees of freedom of the system), shaded areas 2-D areas of uncertainty of points. **(b)** The subsets of grid points defining the intersection S and which are used to compute the corresponding centers of weight, representing the best estimates values of the unknown coordinates (red triangles), summarized in Table 8.

In order to confirm that the previous result was significant and representative of the efficiency of the TOPINV method to solve a wide range of underdetermined systems of equations, a variation of Example 2 is analysed. A rhomb 1-2-3-4 with known coordinates is assumed. From these coordinates the side lengths are computed and white noise (*σ*_j_ = ±4 mm) was added in order to form surrogate measurements of side lengths. The coordinates *x*_*1*_, *y*_*1*_ and *y*_*3*_, *y*_*1*_ = *y*_*3*_ were assumed known, so that an under-determined system with configuration defect was formed (Figure 8). Data are summarized in Table 7. The system of observation equations are as in section “Case study 2: a 2-D nonlinear geodetic/geometric problem”, but for *j* = 1,2,…,n = 5 unknown variables and *i* = 1,2,…,m = 4 measurements/equations.

**Table 7:** **Coordinates and synthetic measurements of distances in the examined 2-D network for case study 3**

Fixed (known) coordinates (m)		Reference (unknown) coordinates (m)		Synthetic measurements (m)	
*x*_1_	100.000	*x*_2_	150.000	ℓ_1_	70.7067
*y*_1_	100.000	*y*_2_	150.000	ℓ_2_	70.7127
*y*_3_	100.000	*x*_3_	200.000	ℓ_3_	70.7137
		*x*_4_	150.000	ℓ_4_	70.7097
		*y*_4_	50.000		

#### SVD solution

The same process as in “Case study 2: a 2-D nonlinear geodetic/geometric problem” was followed using approximate coordinates . Results are summarized in Table 8 and indicate that  is an unbiased and precise estimator of *x* because  was also an unbiased and precise estimator of *x*.

**Table 8:** **Comparison of the TOPINV and SVD solutions with the reference values for Case study 3**

Reference coordinates		TOPINV ( ***k*** =1.25)		SVD
			± ***σ***	
*x*_2_	150.000	150.000	0.001	150.003
*y*_2_	150.000	150.000	0.001	149.991
*x*_3_	200.000	200.006	0.003	200.015
*x*_4_	150.000	150.002	0.002	150.005
*y*_4_	50.000	50.003	0.002	50.006

Values in meters.

#### TOPINV solution

The methodology followed is similar to that in the previous Case Study, for a 5-D grid providing the additional information that possible values of coordinates are in a range of ±3 cm around the reference values (see Table 9). The solution obtained for *k* = 1.25 is summarized in Table 8 and is again very accurate and precise. In Figure 8b is shown in symbolic visualization the final grid points in G and the best estimated coordinates.

**Table 9:** **Details of the grid used in the TOPINV inversion for case study 3**

Coordinates	Reference coordinates	Grid boundaries (m)	Spacing (mm)	Grid points	Total grid points in G
*x*_2_	150.000	149.970–150.030	1.0	61	61^5^ = 844, 596, 301
*y*_2_	150.000	149.970–150.030		61	
*x*_3_	200.000	199.970–200.030		61	
*x*_4_	150.000	149.970–150.030		61	
*y*_4_	50.000	49.970–50.030		61	

### Case study 4: a 9-D non-linear geophysical problem

We examine a common problem in geophysics-seismology-geodesy, the modelling of a seismic fault from observations of displacements of ground stations, derived from the comparison of pre- and post-seismic coordinates, usually on the basis of GPS observations. Seismic faults are defined by 9 parameters constraining their location and kinematics, and certain highly non-linear equations permit to relate the fault characteristics with surface deformation at a selected point on the ground surface (Okada 1985). Because of the complexity of the system of equations and the large number of variables defining a fault (nine variables), fault modelling is usually based on forward analysis (e.g. Feigl and Dupre 1999).

On the basis of two examples it is shown that the TOPINV algorithm can invert an underdetermined system of equations deriving from GPS observations and certain a priori constraints for the fault characteristics and define the fault.

**Figure 9:**
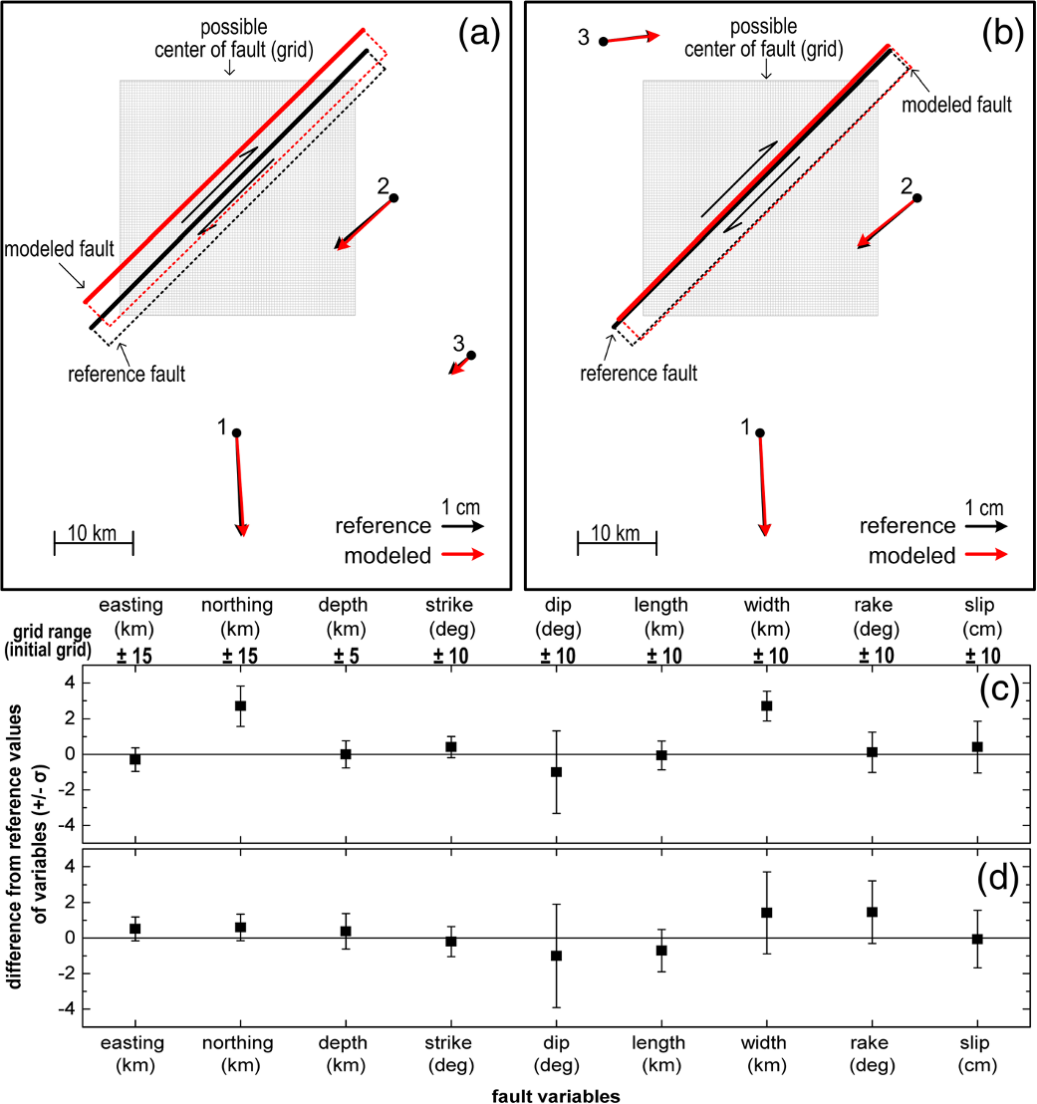
**Two cases of modelling of seismic faults with TOPINV algorithm.** The inversion was basedon limited GPS observations using a priori information (constraints to the fault characteristics derived from geological/seismological information). Top **(a)**, **(b)**: two different cases of oblique strike slip faults. Reference and modelled faults and displacement vectors are shown. Solid lines indicate their surface trace and rectangulars their projection on the surface. Observation stations are marked with numbers. Shaded rectangles indicate the likely position of the centre of the fault. Bottom **(c)**, **(d)**: Deviations of estimated values from their reference values (zero lines) and their 1-*σ* standard deviations for each case respectively. Units for each variable are marked on the each variable, also is shown the a priori assumed possible range of values, used to form the initial grid.

The technique adopted is the following: A certain reference fault is assumed, and from this fault, the predicted reference displacements are computed and regarded as observations; each point (station) contributes with one observation for each coordinate. In each of the two cases, an underdetermined system with 9 unknowns and 3×2 = 6 observations is hence formed. Some reasonable a priori constraints for the fault (a range of possible values for each variable) are then made, shown in Figure 9c,d (for instance that this centre is located somewhere in the rectangular of Figure 9a or b), and then, the TOPINV algorithm is applied. Modelled values (solution) of the fault characteristics are shown. The differences between reference (real) and modelled values, as well as their standard deviations are shown in Figure 9c, d. It is evident that modelled values are statistically non different from the reference values mostly at the 66% significance level.

These estimates were compared with the corresponding reference values and the results for both study cases are summarized in Figure 9. This Figure indicates that bias in results (estimates) is minimum, for in both cases estimates are usually within 1-*σ* and only in a few cases within 2-*σ* from the reference (true) values.

It must be noticed, that the TOPINV algorithm was not applied in a single step, because the range of possible values of the 9 parameters is large. For this reason, and in order to avoid a huge grid (>10^9^ points) delaying computations, the algorithm was applied first for the grid G shown in Figure 9, but with a small number of points (large spacing between grid points). This permitted to identify a compact, convex space *S* which contains the solution. This process was repeated with a new grid G* covering a smaller 9-D space than the initial grid G (G* ⊂ G) but with finer resolution around *S*, and a new space S* was computed. This process was repeated four times, and the final solution *S* was identified, and the estimates of the 9 variables, along with their variances, were computed.

## Discussion

Any underdetermined system is satisfied by an infinite number of solutions, and various sampling techniques can identify some of these solutions. The advantage of SVD is that it offers minimum norm solutions (Strang 2003), the accuracy of which (i.e. their distance from the “real” solution, see Mikhail 1976) depends on the initial conditions; if a good approximation of the unknowns is made, precise and accurate solutions are obtained, as is highlighted in Example 2.

The main limitation of SVD approach is that it requires linear equations and inversion of matrices, and this is not possible in highly non-linear systems, for instance in fault and magma source modelling using surface displacement data. Problems of this type are used on the basis of sampling-based approaches (Pedersen et al. 2003), solutions at steps, with up to two variables solved at each step (Feng and Newman 2009), or forward modelling (Feigl and Dupre 1999; for a discussion see Saltogianni and Stiros 2013). TOPINV (or TGS sensu Harvey 2013) offers the opportunity to solve such problems using a deterministic topological, quasi-deterministic approach, based on simple, forward calculations only, avoiding matrix inversion in the n-D space. In particular, the optimization factor *k* in Eq. (5) permits to identify an optimal solution, a minimum norm solution, not trapped in local minima (see Saltogianni and Stiros 2012a). Hence it offers an algorithm not subject to the limitations of the various sampling-based (mostly Monte-Carlo) approaches (Li 2009).

In reality, what this method permits is to fully exploit the structural and topological constraints existing in each system and imposed by the a priori external information and to identify an n-D closed space containing all possible solutions. This closed space is approximated by a set of gridpoints in R^n^ and their centre of weight defines an optimal solution, compatible to the SVD minimum norm-solution, where this is possible (Case studies 2, 3, 4). Hence the population of possible solutions of the system is at first determined with a quasi-deterministic approach, as intersecting loci, and then the optimal solution is determined using a simple and efficient stochastic approach.

The concept of intersecting geometric loci, on which the proposed method is based, is of course not new, and has been widely used. For instance, intersections of circles, planes, spheres, tori are also used to determine positioning in robotics (Sokolov and Xirouchakis 2006; Ren and Hong 2009), while sources of sound can be defined as intersections of hyperboloids (Hardin et al. 2005).

The use of additional, a priori information for the solution is also not new, but TOPINV permits to exploit this information in a different way: as topological constraints to build-up grid G, while in the past this information was used either for the linearization of equations or the formation of additional equations (Brunner 1979; Prescott 1981; Matsu’ura and Hirata 1982; Jackson and Matsu’ura 1985; Usai 2003; Kampes and Hanssen 2004;. Kotsakis 2012). Obviously this approach simplifies computations and permits to identify the closed space of possible solutions, as is highlighted in section “Methodological approach” and Figure 5.

Obviously, the degrees of freedom of the system (number of equations required for a fully determined equation), the quality of observations (measurements) and the selection of the grid G influence (better control) the quality of the final TOPINV solution. Still, as the case study of “Case study 4: a 9-D non-linear geophysical problem” indicates, the method seems suitable for very non-linear systems and systems with a relatively large number of unknown variables.

The overall approach is possible because it fully exploits the capabilities of modern computers for searches in large grids (with ≥ ~10^8^ points, see Tables 2, 5, and 9). Much larger grids, however, should be avoided, and the analysis should be made in steps, keeping the number of grid points below a certain threshold for common computers; larger grids with lower density at first, gradually leading to smaller, denser grids. This process permits to identify different clusters of solutions, i.e. different solutions, for each of which a different n-D space *S* should be identified.

### Limitations

So far it was assumed that a solution in the underdetermined system exists. Clearly, the quality of a solution, even the possibility of a solution depends on the a priori conditions. This can be highlighted in Figure 5. In Figure 5c the intersection of the space defined by internal constraints (a ring, part of which is shown) and of the a priori conditions (rectangular) is small and permits a clear solution, the precision of which increases with the decrease of the dimensions of the intersection (i.e. the number of its grid points). If the a priori conditions are somewhat vague, simulated by a square around the ring representing the internal constraints (Figure 5a), the intersection is identified with the ring, and does not lead to a closed space and a solution. In such cases, a stochastic geometric locus, corresponding to the mean radius of the ring can only be computed. In the n-D space this situation corresponds to a torus or a toroid.

## Conclusions

The topological inversion algorithm (TOPINV or TGS), initially proposed for the solution of redundant systems of highly non-linear equations was used for certain cases of “free-net adjustments”, i.e. the solution of a certain type of under-determined type of systems of equations by Harvey (2013). Evidence presented above indicates that this algorithm can be successfully used for the solution of a wide range of under-determined problems, such as those found in geophysics (elastic dislocation modelling of a fault, see “Case study 4: a 9-D non-linear geophysical problem”).

This algorithm fully exploits the power of modern computers and the a priori information (constraints) available for most underdetermined systems and can lead to a minimum-norm solution, without the need of matrix inversions.

## Notation

Bold characters indicate vectors

n, m: number of unknowns and of observations

*x*: variable

: true value

: approximate value

: TOPINV best estimate,

: SVD best estimate

***υ***, **ϵ**: errors

**ℓ**: measurements

*δ****x***: difference between approximate and true value

G: n-D grid

*S*: subset of G bounding the solution

*A*: design matrix in a linear system of observations

*J*: Jacobian of the system of non-linear equations
